# Impaired Cognitive Functioning in Cochlear Implant Recipients Over the Age of 55 Years: A Cross-Sectional Study Using the Repeatable Battery for the Assessment of Neuropsychological Status for Hearing-Impaired Individuals (RBANS-H)

**DOI:** 10.3389/fnins.2018.00580

**Published:** 2018-08-24

**Authors:** Annes J. Claes, Paul Van de Heyning, Annick Gilles, Anouk Hofkens-Van den Brandt, Vincent Van Rompaey, Griet Mertens

**Affiliations:** ^1^Department of Otorhinolaryngology, Head and Neck Surgery, Antwerp University Hospital, Antwerp, Belgium; ^2^Experimental Lab of Translational Neurosciences and Dento-Otolaryngology, Faculty of Medicine and Health Sciences, University of Antwerp, Antwerp, Belgium; ^3^Department of Human and Social Welfare, University College Ghent, Ghent, Belgium

**Keywords:** cognition, cochlear implantation, RBANS-H, older adults, profound hearing loss, RBANS

## Abstract

**Primary Objective:** To compare cognitive functioning among experienced, unilateral cochlear implant (CI) recipients and normal-hearing (NH) controls by means of the Repeatable Battery for the Assessment of Neuropsychological Status for Hearing-impaired individuals (RBANS-H).

**Methods:** Sixty-one post-lingually and bilaterally severely hearing-impaired CI recipients (median age: 71.0, range: 58.3 to 93.9 years) with at least 1 year of CI experience (median: 12.4, range: 1.1 to 18.6 years) and 81 NH control participants (median age: 69.9, range: 50.1 to 87.1 years) took part in this cross-sectional study. The RBANS-H was performed, as well as an audiometric assessment, including best-aided speech audiometry in quiet (monosyllabic words) and in noise (Leuven Intelligibility Sentences test).

**Results:** The RBANS-H performances of the CI recipients (mean: 88.1 ± 14.9) were significantly poorer than the those of the NH participants (mean: 100.5 ± 13.2), with correction of age, sex, and education differences (general linear model: *p* = 0.001). The mean difference, corrected for the effects of these three demographic factors, was 8.8 (± 2.5) points. Additionally, in both groups, a significant correlation was established between overall cognition and speech perception, both in quiet and in noise, independently of age.

**Conclusion:** Experienced, unilateral CI recipients present subnormal cognitive functioning, beyond the effect of age, sex and education. This has implications for auditory rehabilitation after CI and may highlight the need for additional cognitive rehabilitation in the long term after implantation. Long-term prospective and longitudinal investigations are imperative to improve our understanding of cognitive aging in severely hearing-impaired individuals receiving CIs and its association with CI outcomes.

## Introduction

Recently, large cross-sectional studies have established a persisting correlation between hearing loss and poorer cognitive performances in the older population (Lin, 2011; Lin et al., 2011a; Quaranta et al., 2014; Bush et al., 2015; Wayne and Johnsrude, 2015). Furthermore, evidence from several longitudinal studies pointed out that baseline hearing loss is associated with accelerated cognitive decline and incident dementia over time (Lin et al., 2011b, 2013; Gallacher et al., 2012; Gurgel et al., 2014). More specifically, in a prospective, longitudinal study including nearly 2000 community-dwelling older adults aged 70 to 79 years, Lin et al. (2013) found that this association was independent of demographic and cardiovascular risk factors, such as age, sex, education, and stroke history. Individuals with hearing loss, defined as a pure tone average of hearing thresholds at 0.5 to 4 kHz above 25 dB HL in the better ear, had a 30 to 40% accelerated rate of cognitive decline and a 24% increased risk of incident cognitive impairment over a 6-year period compared to individuals with normal-hearing (NH). The causal mechanism underlying the link between hearing loss and cognitive decline, however, is still a matter of debate (Wayne and Johnsrude, 2015; Roberts and Allen, 2016).

One hypothesis is that a common cause, for instance widespread neural degeneration, generates the decline in both hearing and cognition. This is the *common cause hypothesis* (Lindenberger and Baltes, 1994; Baltes and Lindenberger, 1997). An alternative is the *cognitive load on perception hypothesis* (Arlinger et al., 2009; Wayne and Johnsrude, 2015). According to this hypothesis, cognitive decline results in hearing loss, as it reduces the cognitive resources that are available for auditory perception (Pichora-Fuller, 2003; Wayne and Johnsrude, 2015). Another explanation involves a causal relationship in the opposite direction; hearing loss leads to cognitive decline that is either permanent in the case of the *sensory deprivation hypothesis*, or potentially reversible and remediable in the case of the *information degradation hypothesis* (Pichora-Fuller, 2003). As regards the *information degradation hypothesis*, older hearing-impaired adults have to compensate for impoverished auditory input through increased reliance on cognitive resources. As the perceptual difficulties cascade *upwards*, more cognitive resources are diverted to perception and this, in turn, reduces the available cognitive resources for other tasks. This eventually results in compromised cognitive performance. This view has gained considerable support from several studies pointing out that cognitive performances decrease when the speech signal is degraded, for instance by introducing background noise (e.g., McCoy et al., 2005; Piquado et al., 2010). A crucial implication of this information degradation hypothesis is that the impairment in cognitive performance could be alleviated and the onset of dementia postponed by auditory interventions (Pichora-Fuller, 2003; Arlinger et al., 2009; Wayne and Johnsrude, 2015). Therefore, many studies have investigated the effect of hearing aids on cognition in older hearing-impaired adults. These studies, however, generated conflicting results.

For instance, Acar et al. (2011) found a decrease of depressive signs and an increase of cognitive functions on the Mini-Mental State Examination (MMSE) (Folstein et al., 1975) after using hearing aids for 3 months. In this prospective, single-arm interventional study 34 older adults over the age of 65 years with a moderate to severe hearing impairment were included. Choi et al. (2011) also demonstrated an improvement in speech-related cognitive function, assessed by means of a computerized visual verbal learning test, after 6 months of hearing aid use in 18 older participants. In the control group no change was present between the baseline measurement and the second measurement 6 months later. In addition, Amieva et al. (2015) followed-up 3,670 individuals aged 65 years and over during an exceptionally long period of 25 years. The results indicated that the cognitive decline, as measured on the MMSE, in hearing-impaired adults without hearing aids was accelerated compared to participants who reported normal hearing. In contrast, the cognitive decline in hearing-impaired adults who did use hearing aids did not differ from the control participants. On the other hand, van Hooren et al. (2005) did not find any improvement in cognitive performance after a follow-up of 12 months among a group of older adults receiving hearing aids compared to a group of control participants with an equivalent hearing impairment but without hearing aids. Unexpectedly, the intervention group even had poorer performance on one measure of the Stroop color-word test than the control group 1 year after the hearing aids were fitted. Mixed results may be explained by the wide variety of study designs (e.g., with or without control group), outcome measures, and the selection and characteristics of participants.

Only very recently, the effect of auditory rehabilitation by means of a cochlear implant (CI) on cognition in older profoundly hearing-impaired adults became a subject of research. In a pioneering study with 94 participants, Mosnier et al. (2015) found that intervention by means of cochlear implantation in older adults was associated with improvements in preoperatively impaired cognitive capabilities after six and 12 months of CI use. These results were confirmed by Cosetti et al. (2016) in a study population of seven women and by Castiglione et al. (2016) in a group of 15 individuals, receiving a CI. The authors suggest that correction for hearing loss by the use of CIs may have a protective effect against reduced cognitive function.

Although considerable research has been devoted to the effects of hearing solutions on cognitive functioning in older, hearing-impaired adults, it remains unexplored whether severely hearing-impaired individuals with a CI perform age-expected in terms of cognition or not. Therefore, the aim of the present study was to assess cognitive functioning in CI recipients with at least 1 year of CI experience. Experienced CI users above the age of 55 years were included. Cognition was evaluated by means of the *Repeatable Battery for the Assessment of Neuropsychological Status for Hearing-impaired individuals* (RBANS-H) (Claes et al., 2016). This test is a modification of the *Repeatable Battery for the Assessment of Neuropsychological Status* (RBANS) (Randolph, 1998), a widely used and well-accepted neuropsychological test battery for the clinical diagnosis and tracking of dementia and mild cognitive impairment. The RBANS-H scores obtained in the CI group were compared to the RBANS-H scores obtained in a NH control group, with correction for age, sex, and education differences. As Lin et al. (2013) reported a linear association of rates of cognitive decline with the severity of an individual’s hearing loss, one may hypothesize that CI users, who are all severely to profoundly hearing-impaired, are particularly prone to higher rates of cognitive decline and cognitive impairment and may, therefore, present lower cognitive performances than expected based on age. However, if auditory rehabilitation by means of a CI indeed protects against reduced cognitive functions, which is suggested by Mosnier et al. (2015), Castiglione et al. (2016), and Cosetti et al. (2016), then age-normal cognitive performances would be expected in this population. In addition, the association between cognitive performance on the one hand and demographic factors and hearing capabilities on the other hand was explored in both groups separately.

## Materials and Methods

### Study Design

In the present cross-sectional study, both the CI recipients and the NH control participants were assessed once, under the supervision of a single experienced, Good Clinical Practice certified audiologist (Master of Science). Examination consisted of a cognitive and an audiological assessment, including best-aided speech audiometry in quiet and in noise. The complete assessment took one to one and a half hours.

### Participants

#### CI Recipients

Participants from the Otorhinolaryngology, Head and Neck Surgery department of the Antwerp University Hospital (UZA), Belgium were invited to participate in the study, based on the CI registry database. Out of approximately 1000 patients registered in this database, 145 were found eligible according to the following criteria: the person (1) was at least 55 years old, (2) was post-lingually, bilaterally and severely to profoundly hearing-impaired, (3) had received a CI unilaterally in accordance to the Belgian national reimbursement criteria (i.e., the person had a mean preoperative hearing loss at 0.5, 1, and 2 kHz of at least 85 dB HL at the better ear), (4) had at minimum 1 year of experience with the CI and (5) was a daily CI user. Patients were excluded from the study if they were unable to complete the test protocol due to uncorrected vision impairment or other impairments. Sixty-one participants (30 males, 31 females) were included. The median age of the participants was 71.0 years, ranging from 58.3 to 93.9 years and the median age at implantation was 62.5 years (range: 44.8 to 87.9 years). CI experience ranged from 1.1 to 18.6 years (median: 12.4 years). The participants had had 6 to 20 years of formal education with a median of 11 years. All participants were tested with their normal everyday processor settings, as fitted by an experienced audiologist. Fifty CI recipients (82%) used their unilateral CI without contralateral hearing aid (30 right, 20 left), whereas 11 participants (18%) used a contralateral hearing aid (9 hearing aid right, CI left and 2 hearing aid left, CI right).

#### Control Group

A population-based sample of 103 participants aged 50 to 89 years was recruited by means of the population registries, made available by the local city councils in southern Antwerp (Belgium), by advertisements in the hospital and by approaching friends, family and acquaintances, put in by several colleagues and students working in the hospital. Participants were excluded if any of the following criteria was not met: participants (1) were between the age of 50 and 89 years old (50 and 89 included), (2) had normal thresholds at 0.250 up to 8 kHz, based on age and sex, as defined by the BS 6951:1988, EN 27029:1991, and ISO 7029-1984 standards, (3) had no history of any neurological disease (e.g., dementia, Parkinson’s disease, cerebrovascular accident, etc.) and (4) had no uncorrected vision impairment. After a screening phase, in which hearing was examined by means of pure-tone audiometry and the medical history was questioned, 81 participants (39 males and 42 females) were eventually included (median age: 69.9 years, range: 50.1 to 87.1 years). The NH participants had had 8 to 22 years of formal education with a median of 14 years. The majority of the NH controls did not use hearing aids (*n* = 76, 94%). Yet, five participants (6%) used bilateral hearing aids, although their hearing was within the normal range for their age and sex.

### Outcome Measurements

#### Cognitive Assessment

Cognitive function was assessed by means of the RBANS-H (Claes et al., 2016). The RBANS-H is a modification of the RBANS (Randolph, 1998), which is a neuropsychological test battery for the clinical diagnosis and tracking of dementia and mild cognitive impairment. A major advantage of the RBANS is that it yields one total score of cognition, which can be converted to an age-corrected standard score with a mean equal to 100 and a standard deviation equal to 15. It consists of 12 subtests, assessing five cognitive domains. The first domain, *Immediate memory*, consists of the subtest List learning and Story memory. *Visuospatial/constructional* capabilities are assessed using a Figure copy and Line orientation task. The third domain is *Language*. This comprises Picture naming and Semantic fluency. *Attention* is evaluated using a Digit span and a Coding task. Finally, *Delayed memory* includes the List recall, List recognition, Story recall and Figure recall subtests. The RBANS is provided with normative data for the following age categories: 12–13, 14–15, 16–19, 20–39, 40–49, 50–59, 60–69, 70–79, 80–89 years. Due to its short administration time (approximately 20 to 30 min), the test is suitable for neuropsychological testing in a clinical setting (Randolph et al., 1998; Appels and Scherder, 2010; Karantzoulis et al., 2013). The RBANS is a complete cognitive test battery with a good sensitivity to change, which is also capable of differentiating between different levels of *normal* cognition. This greatly contrasts with cognitive screening tools such as the MMSE (Folstein et al., 1975) and the Montreal Cognitive Assessment (MoCA) (Nasreddine et al., 2005), which present ceiling effects in good-performing individuals.

Similarly, the modified RBANS-H is a neuropsychological test battery for examining cognitive function specifically in hearing-impaired individuals. As examinees with a hearing impairment are at a disadvantage if the RBANS is administered in a standardized manner with oral instructions and item presentation, developing a cognitive test battery for hearing-impaired subjects was necessary. In contrast to the RBANS, in which the instructions are given orally, the RBANS-H provides written instructions, presented on a PowerPoint presentation, in combination with the standard oral instructions. Furthermore, four of the 12 subtests that are solely orally presented in the RBANS, are adjusted in the RBANS-H by providing a combination of visual and auditory stimulation. An extensive and detailed description of the RBANS-H and the modifications can be found in Claes et al. (2016).

#### Audiological Assessment

Speech audiometry in quiet and in noise were performed according to the Minimal Outcome Measurements (Kleine Punte and Van de Heyning, 2013). All the audiological measurements were performed using a two-channel Interacoustics AC-40 audiometer in a sound-treated booth. For free field measurements, a loudspeaker was positioned at one-meter distance in front of the participant, at ear level (0° azimuth). Speech audiometry in quiet and in noise were performed in free field, in the best-aided condition. For CI recipients the best-aided condition was either with CI only or with CI in combination with contralateral hearing aid. For the NH group, the best-aided free field speech audiometry was performed either unaided (when the participant did not use any type of hearing aid in daily life) or with hearing aids.

##### Speech audiometry in quiet

For the CI recipients, speech audiometry in quiet was performed at 65 dB SPL in free field, according to an international protocol established for the follow-up of CI recipients in the hospital (Kleine Punte and Van de Heyning, 2013). In the NH control participants, the speech intelligibility scores at 65 dB SPL in the NH group were on average 97%, with only three participants obtaining a score less than 90%. Because of the lack of variability in performance at 65 dB SPL among the control group, the speech reception threshold (SRT), or the level at which 50% of phonemes was correctly received, was used instead. The speech materials used were the Dutch open-set NVA lists developed by the Nederlandse Vereniging voor Audiologie (NVA) or Dutch Society for Audiology (Wouters et al., 1994). Each list consists of 12 monosyllabic words (consonant-vowel-consonant) of which the first one is a training item. The speech recognition score is the percentage of correctly identified phonemes.

##### Speech audiometry in noise

Speech reception in noise was assessed in free field using the Leuven Intelligibility Sentences Test (LIST) in an adaptive procedure (van Wieringen and Wouters, 2008). This Dutch speech material consists of 35 lists of 10 sentences and has been developed and validated for use with severely hearing-impaired individuals and CI recipients. Speech-weighted stationary noise, based on the long-term average speech spectrum of the sentences, was presented at a fixed level of 65 dB SPL. The starting level of the speech was also 65 dB SPL, but the level was altered in steps of 2 dB depending on the response of the participant. If all the keywords of a given sentence were repeated correctly, the level of the speech was decreased by two decibels. The level of the speech was increased by 2 dB if the keywords were not correctly repeated. The SRT was calculated as the mean level of the last five sentences and the level of the imaginary 11^th^ sentence. The lower, or the more negative, the SRT (dB SNR), the better speech in noise is perceived.

### Ethics Statement

This study was conducted in accordance with the recommendations of the ethics committee of the Antwerp University Hospital/University of Antwerp. The protocol for the CI recipients was approved on August 10^th^, 2015 (Protocol No. 15/25/268) and the one for the NH control participants was approved on November 21^st^, 2016 (Protocol No. 16/43/450). All participants gave written informed consent in accordance with the Declaration of Helsinki prior to participation.

### Data Management and Statistical Methods

Data were stored in OpenClinica LLC (Waltham, MA, United States), a password protected online database for electronic data capture and data management developed for clinical research. IBM SPSS Statistics (IBM; Armonk, NY, United States) was used for the statistical analyses. Descriptive statistics were used to summarize the distribution of RBANS-H total scores. In addition, it was investigated whether the RBANS-H total scores differed significantly between both groups (CI recipients versus control participants), after correction for age, sex, and education differences. This was done by means of a general linear model, with RBANS-H scores as dependent variable, group and sex as independent fixed factors, and age and education (i.e., the number of years of formal education starting from the age of 6) as covariates. Also the estimated marginal mean difference (NH group minus CI group) was calculated, i.e., the mean difference on the RBANS-H total score between both groups, corrected for age, sex, and education. A significance level of α = 0.05 was applied. Descriptive statistics were also performed to summarize the speech in quiet and speech in noise scores. In addition, correlations were calculated to assess the association between the RBANS-H total scores and (1) age, (2) number of years of experience with CI (CI experience), (3) education, (4) speech perception performance in quiet (CI group: percentage correct at 65 dB SPL and NH group: SRT) and (4) speech perception performance in noise (SRT). Since most variables were not normally distributed, Spearman correlations were calculated for all correlations. Due to the explorative character of the correlational analyses, no correction for multiple testing was applied.

## Results

**FIGURE 1:**
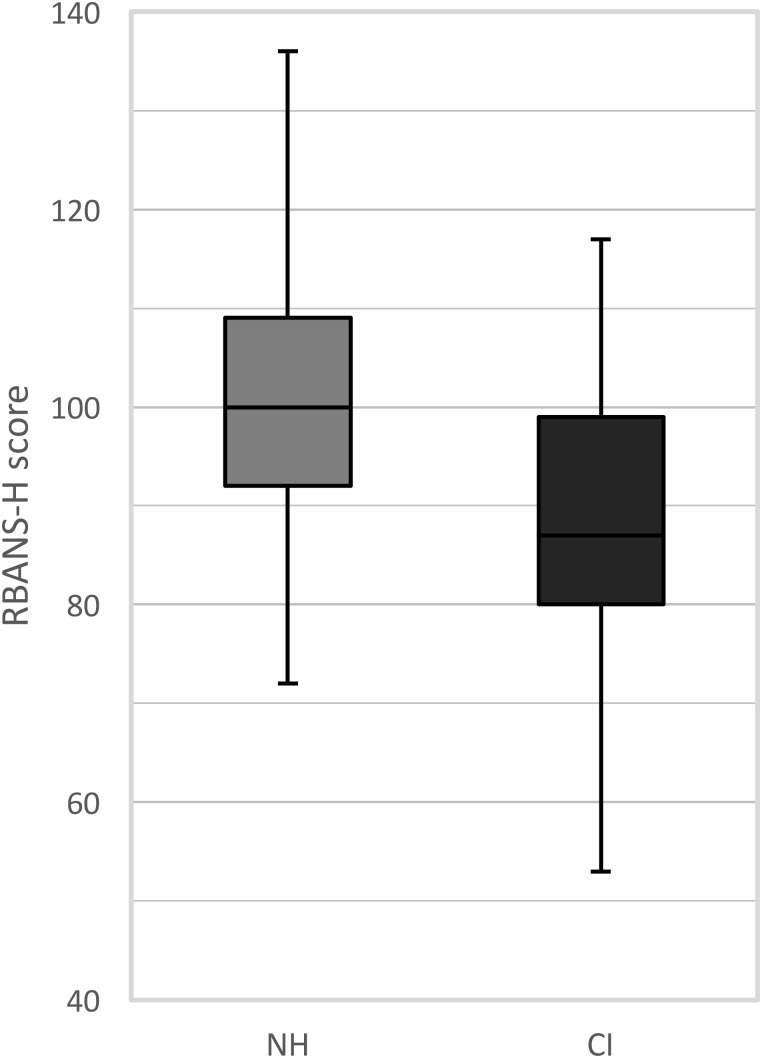
Boxplots of the RBANS-H scores for the NH group (normal-hearing, gray boxplots) and the CI group (cochlear implant, black boxplots). The boxplots represent the minimum, 1^st^ quartile, median, 3^rd^ quartile and maximum of the RBANS-H total score.

The RBANS-H results are presented for the CI recipients and the NH control group separately in **Figure 1**. Among the CI recipients, the mean RBANS-H total score was 88.1 (± 14.9) (median: 87.0, range: 53 to 117). The mean total score among the control group was 100.5 (± 13.2) (median: 100.0, range: 72 to 136). A general linear model pointed out that the CI recipients performed significantly poorer than the NH participants, after controlling for age, sex, and education differences (*p* = 0.001). The estimated marginal mean difference of the RBANS-H total score between both groups was 8.8 (± 2.5) points, taking age, sex, and education into account.

As expected, the RBANS-H scores, which are age-corrected, were not significantly correlated to age in the NH group (ρ = -0.092, *p* = 0.414), nor was a significant correlation observed in CI recipients (ρ = -0.249, *p* = 0.053). CI experience did not present an association with RBANS-H score, among the CI recipients (ρ = 0.129, *p* = 0.323). With regard to education, the correlation to RBANS-H score depended on the group. In the control group, no association was demonstrated (ρ = 0.095, *p* = 0.398), but in the CI recipients a significant positive association was found (ρ = 0.332, *p* = 0.009).

For the CI recipients, the mean speech intelligibility score in quiet at 65 dB SPL was 73 (± 17.6%) (median: 79%, range: 21 to 97%). In the NH control participants, the speech intelligibility scores at 65 dB SPL were on average 97%, with only three participants obtaining a score less than 90%. Due to the lack of variability in performance at 65 dB SPL, the SRT was used instead for the NH participants. The mean SRT in quiet was 30 (± 9.1) dB SPL (median: 28 dB SPL, range: 14 to 51 dB SPL). In both groups, better speech intelligibility in quiet correlated with better RBANS-H scores (CI: ρ = 0.313, *p* = 0.014 and NH: ρ = -0.256, *p* = 0.021). This association remained significant with correction for age (CI: ρ_part_ = 0.272, *p* = 0.035 and NH: ρ_part_ = -0.269, *p* = 0.016). Among the NH participants, the correlation between SRT and RBANS-H scores was negative, as a lower SRT, i.e., better speech perception in quiet, was associated with better cognitive performances.

Among the CI recipients, the mean SRT in noise was +8.1 (± 7.1) dB SNR (median: +5.5 dB SNR, range: -2.0 to +20.0 dB SNR). The mean SRT in noise in the control group was -3.1 (± 2.5) (median: -3.7 dB SNR, range: -7.0 to 3.0 dB SNR). SRT in noise demonstrated a significant association with RBANS-H score in both groups (CI: ρ = -0.354, p = 0.005 and NH: ρ = -0.354, p = 0.001). This association remained significant with correction for age (CI: ρ_part_ = -0.293, p = 0.023 and NH: ρ_part_ = -0.362, p = 0.001). The correlation between RBANS-H total score and speech in noise is negative, as a lower SRT, i.e., better speech perception in noise, indicates better performance on the RBANS-H.

## Discussion

The present study aimed at investigating cognitive performance by means of the RBANS-H in experienced CI users above the age of 55 years, compared to a NH control group with correction for age, sex, and education differences. In addition, the correlations between cognitive capabilities on the one hand and demographic factors and speech perception performance on the other hand were explored in both groups.

The results with the RBANS-H demonstrated that overall cognitive functioning among older adults is significantly poorer in CI recipients than in NH individuals, regardless of age, sex, and education. The mean difference in RBANS-H total score between both groups, corrected for the effect of these three demographic factors, was 8.8 (± 2.5) points. Since the RBANS-H total score is scaled to a normal distribution with a mean of 100 and a standard deviation of 15, a corrected difference of 8.8 points indicates that the CI recipients perform approximately 0.6 standard deviations lower than the NH peers. Therefore, the observed difference is not only statistically significant, but also clinically relevant. In conclusion, cognitive functioning in severely to profoundly hearing-impaired CI recipients appeared to be subnormal, regardless of age, sex, and education differences.

These findings contradict with the outcomes of Castiglione et al. (2016), who demonstrated no significant difference on the MoCA between 20 NH older listeners and 15 older CI users with 1 year of CI experience. Yet, several aspects of the MoCA, as opposed to the RBANS, may account for the lack of observed difference in the study of Castiglione et al. (2016). The MoCA is a cognitive screening tool to detect mild cognitive impairment and takes approximately 10 min to administer (Nasreddine et al., 2005). It is less sensitive to change than the RBANS and is likely to present ceiling effects in cognitively normal individuals. These characteristics, together with the smaller sample size, may have obscured a possible difference between the CI recipients and the NH participants in the study of Castiglione et al. (2016).

The obtained findings in the present study are expected based on the study of Lin et al. (2013), in which an association is reported of rates of cognitive decline with the severity of an individual’s hearing loss: persons with a more severe hearing loss tend to be at greater risk of accelerated cognitive decline and cognitive impairment than those with a less severe or no hearing impairment. This is also in line with the results of Baltes and Lindenberger (1997), in which poorest intellectual abilities were observed among the hearing-impaired individuals, as compared to the NH individuals, in 70- to 103-year-olds. Both aforementioned studies comprised a large, population-based sample of participants, including persons with severe hearing losses, but not exclusively. As the present study only includes severely to profoundly -and no mildly or moderately- hearing-impaired participants, the cognitive deviation from the norm may, indeed, be more pronounced. A protective effect of cochlear implantation against accelerated cognitive decline, as is suggested by Mosnier et al. (2015); Castiglione et al. (2016), and Cosetti et al. (2016), can be neither confirmed nor ruled out based on the present cross-sectional results. Yet, if cochlear implantation indeed positively affects cognition in older adults, then this effect is, in all probability, not big enough to catch-up with the NH peers and to maintain a normal cognitive aging process, as is indicated by the current findings.

Since the cognitive test battery, the RBANS-H, was modified for the hearing-impaired by providing audiovisual stimulation, the hearing impairment itself was less likely to have placed the participants at a disadvantage during test administration. Alternatively, the finding that the CI recipients demonstrated overall poorer cognitive performance compared to the NH participants ought to be explained by the fact that a CI provides someone who is deaf or severely hearing-impaired with a sense of sound, but does not restore hearing completely. Furthermore, the vast majority (79%) of these bilaterally, severely hearing-impaired participants only receive auditory input through their unilateral CI, as they do not currently use a contralateral hearing aid. This makes the perception of binaural cues extremely hard or impossible, leading to for instance poor localization in the horizontal plane (Grantham et al., 2008). Irrespective of the reasons for this cognitive deviation in older CI recipients, it is clearly pointed out that their cognition is below expectation, based on age, sex, and education. This fact has major implications for auditory rehabilitation after cochlear implantation and should be taken into account to optimize rehabilitation. Moreover, future research should investigate the effects of additional rehabilitative, cognitive training especially customized for hearing-impaired older adults.

Age did not correlate with the RBANS-H scores for either the NH group or the CI recipients. This was expected, since the RBANS-H score incorporates an age correction. The correlation of education to cognition differed along both groups. In the control group, no association was found, whereas, in the CI group, a significant positive correlation was demonstrated. Furthermore, the correlations of speech perception and cognition were examined. In both groups, speech intelligibility in quiet and in noise appeared to correlate with better overall cognitive functioning, irrespective of age.

Hua et al. (2017) also investigated the relationship between speech perception and cognitive performance in CI recipients cross-sectionally. Yet, in contrast to the present study, the 17 included bimodal CI users were younger adults, aged 28 to 74 years. They performed two speech recognition tests, one in quiet and one in noise, and two cognitive tests, the Reading Span Test (RST) and the Trail Making Test (TMT) assessing working memory capacity, and processing speed, attention, executive control and task-switching ability respectively. Both the TMT and the RST were found to correlate with some, however not all, of the speech recognition measures. After correction for age however, the speech test in noise was not correlated to any of the cognitive tests, but the speech test in quiet did show a substantial correlation to the RST and the TMT. As speech understanding in noise is considered more cognitively demanding, based on the Ease of Language Understanding (ELU) model (Ronnberg et al., 2013), a stronger link of cognitive performance to speech understanding in noise than to speech understanding in quiet was expected, contradicting both the results of Hua et al. (2017) and the results of the present study. However, the speech understanding test in noise in both studies involved semantically meaningful sentences, whereas in the speech test in quiet, the participants had to repeat short, monosyllabic words. The amount of contextual and semantic information available in the former test was therefore larger than in the latter, making the speech test in noise possibly less cognitively demanding in the case of the Hua et al. (2017) study and equally demanding in the present study compared to the speech in quiet test. In addition, the differences in associations in both studies could be accounted for by the difference in age of the participants, as the association between cognition and speech perception is reported to change with age (Sommers, 1997).

Limitations of the present study involve the cross-sectional design of the study, which is not suitable for making statements about the cognitive evolution of CI patients over time and for determining cause and effect. Therefore, more prospective, longitudinal research is needed to investigate the evolving relationship between cognitive performance and hearing-related capabilities in CI patients and to explore the effects of cochlear implantation on this relationship. Also the impact of other known risk factors of cognitive decline and dementia, such as diabetes, smoking, hypertension, etc. should be taken into account in future research (Barnes and Yaffe, 2011). Furthermore, in the present study participants were not formally tested for reading ability. However, participants were asked prior to testing, whether they could easily read the instructions of the RBANS-H on the screen. In case of suspicion of illiteracy or visual impairments, the participants were excluded from the study. Another limitation is the lack of correction for multiple testing in the case of the correlational analyses. However, these analyses are exploratory in nature and further investigations, either focusing on one aspect of these correlations or including more participants, are necessary to evaluate the association between demographic factors, audiometric capabilities and cognitive performance in CI recipients.

## Conclusion

The present cross-sectional study demonstrated that CI recipients above the age of 55 years present overall poorer cognitive functioning, as measured on the RBANS-H, in comparison to NH peers, independently of age, sex and education. The mean difference, corrected for the effects of these three demographic factors, was 8.8 (± 2.5) points or 0.6 SD, which is both statistically significant and clinically relevant. Additionally, a significant correlation was established in the CI recipients and the NH control group, between overall cognition and speech understanding, both in quiet and in noise, independently of age. The finding that CI recipients above the age of 55 years have impaired cognitive functioning has implications for auditory rehabilitation after cochlear implantation and may suggest the need for additional cognitive rehabilitation in the long term after implantation. Long-term prospective and longitudinal investigations are imperative to improve our understanding of cognitive aging in older severely hearing-impaired individuals receiving CIs and its association with hearing performance and speech understanding.

## Author Contributions

AC, PVdH, AG, AH-VdB, VVR, and GM conceived and designed the study. AC, PVdH, and GM coordinated the study. AC undertook data collection and analysis and drafted the manuscript. GM, AG, and AH-VdB played a role in data collection and analysis. GM, AG, AH-VdB, VVR, and PVdH critically revised the manuscript. All authors read and approved the final manuscript.

## Conflict of Interest Statement

The authors declare that the research was conducted in the absence of any commercial or financial relationships that could be construed as a potential conflict of interest.
